# Microbiological safety and antimicrobial resistance in cashew-based plant-based cheese analogues

**DOI:** 10.1007/s42770-026-02092-7

**Published:** 2026-09-23

**Authors:** Natália Zorrer Dalmina, Amanda Ladeira Toigo, Kauane Rosane Kommers Krebs, Caroline Isabel Kothe, Roberta Fogliatto Mariot, Heryk Motta, Lívia Kmetzsch, Taís Suhre, Vinícius Klain, Ana Paula Guedes Frazzon, Fabiana Quoos Mayer, Jeverson Frazzon

**Affiliations:** 1 Food Science Institute, Federal University of Rio Grande do Sul (UFRGS), Porto Alegre, RS Brazil; 2 Post-Graduation Program in Cellular and Molecular Biology, Biotechnology Center, UFRGS, Av. Bento Gonçalves 9500, Building 43212 - Room 208, Campus do Vale, Porto Alegre, RS 91501-970 Brazil; 3 Department of Microbiology, UFRGS, Porto Alegre, RS Brazil; 4 The Novo Nordisk Foundation Biotechnology Research Institute for Green Transition, Technical University of Denmark, Kongens Lyngby, DK-2800 Denmark

**Keywords:** Microbiological safety, Lactic acid bacteria, Whole genome sequencing, Mobile genetic elements, Resistance genes

## Abstract

The demand for plant-based cheese analogues has increased in recent years, driven both by the growing adoption of vegan and vegetarian diets and by consumers seeking plant-based alternatives to animal-derived products. Their production relies on diverse raw materials and often incorporates fermentation processes involving molds, yeasts, and lactic acid bacteria (LAB). Within this context, ensuring food safety, including the antimicrobial resistance (AMR) aspects, is essential due to the complex microbial communities involved in fermentation. Therefore, this study investigated the presence of bacterial species of potential public health relevance and assessed AMR profiles and genes in plant-based cheese analogues acquired in Brazil. None of the samples tested positive for *Salmonella* spp., coagulase-positive *Staphylococcus*, or *Escherichia coli*, while *Bacillus cereus* was detected in one sample. Two atypical colonies recovered on selective media for *Salmonella* were identified as *Cronobacter malonaticus* and *Delftia acidovorans*. Most samples exhibited high mold and yeast counts, likely associated with fermentation and maturation processes. Among 176 LAB isolates, 11 species were identified by MALDI-TOF, and twenty representative strains were selected for antimicrobial susceptibility testing. Resistance was detected in a limited number of isolates, mainly to ciprofloxacin, tetracycline, and gentamicin, whereas *C. malonaticus* exhibited resistance to penicillin. Whole-genome sequencing was performed on *Lactiplantibacillus plantarum*, *Levilactobacillus brevis*, *Leuconostoc mesenteroides*, *Enterococcus faecium*, and *C. malonaticus*. A total of eight antimicrobial resistance genes were identified, four of which were located on plasmids in *C. malonaticus* and *L. brevis*. These findings suggest that microorganisms associated with plant-based cheese analogues may act as reservoirs of antimicrobial resistance determinants, highlighting the importance of safety assessment and microbiological surveillance of strains intended for technological or probiotic applications.

**Supplementary Information:**

The online version contains supplementary material available at https://doi.org/10.1007/s42770-026-02092-7.

## Introduction

Food safety remains a major public health concern worldwide, particularly in foods that rely on microbial fermentation [1–3]. Fermented foods contain complex microbial communities that contribute to product quality, stability, and sensory characteristics, but may also include undesirable microorganisms introduced through raw materials, processing environments, or post-production contamination [4–8].

In recent years, the market for plant-based cheese analogues has increased substantially due to the growing consumer demand for sustainable, ethical, and health-conscious alternatives to animal-derived products [9]. These products are commonly produced from nuts, legumes, seeds, starches, and vegetable oils, and frequently involve fermentation processes mediated by yeasts, molds, and LAB to improve flavour, texture, shelf life and overall product acceptance [10–14]. Among these microbes, LAB play a central technological role by contributing to acidification, flavour development and inhibition of spoilage microorganisms.

Despite the rapid growth of the plant-based cheese sector, information regarding the microbiological safety of these products remains limited. This is particularly relevant because many products are manufactured by small-scale producers and may not undergo heat treatment or fermentation [15, 16], potentially increasing their susceptibility to contamination by opportunistic or pathogenic microorganisms. Furthermore, the microbial populations present in fermented foods have attracted increasing attention as potential reservoirs of antimicrobial resistance (AMR) determinants [15, 17, 18]. Although LAB are generally recognized as safe and are widely used as starter cultures and probiotics, some strains have been reported to harbor acquired antimicrobial resistance genes (ARGs), especially those associated with mobile genetic elements that may facilitate horizontal gene transfer within microbial communities [5].

The presence of ARGs in food-associated microorganisms does not necessarily represent a direct health risk; however, monitoring these determinants contributes to a better understanding of the potential role of foods in the ecology and dissemination of AMR. Consequently, the assessment of both microbiological safety and AMR profiles has become increasingly relevant in fermented food systems [19].

Given this context, the present study evaluated the microbiological safety of Brazilian cashew-based plant-based cheese analogues and investigated the antimicrobial susceptibility profiles of lactic acid bacteria and bacterial contaminants isolated from these products. In addition, selected isolates were characterized by whole-genome sequencing to assess the occurrence of AMR genes.

## Materials and methods

### Sample collection

Thirteen samples (S1–S13) of Brazilian cashew-based plant-based cheese analogues were acquired from local supermarkets, fairs, and e-commerce platforms (Supplementary Table 1). The sample selection was carried out through searches on networks such as Google, considering products available between August 2023 and January 2024. The samples were stored at 4 °C, and microbiological analyses were performed before the expiry date indicated on the product label.

### Identification of indicator and pathogenic microorganisms

The selection of microorganisms investigated was based on Annex I of Normative Instruction (IN) No. 161, dated July 1, 2022, issued by the Brazilian Health Regulatory Agency (ANVISA), specifically in group 4 (other plant-based products), category (c): Refrigerated tofu, sufu, and similar products [20]. Microbiological analyses included the detection of *Salmonella* spp. and the enumeration of coagulase-positive *Staphylococcus*, *Escherichia coli*, presumptive *Bacillus cereus*, and molds and yeasts. The methods were based on the International Standards Organization (ISO) 6579:2002, ISO 6888-1:1999, ISO 16649-2:2001, ISO 7932:2004 e ISO 21527-1:2008, with adaptations [21].

For each assay, 25 g of the sample was homogenized in 225 mL of buffered peptone water (BPW; Oxoid, Basingstoke, UK). Coagulase-positive *Staphylococcus*, presumptive *B. cereus*, and molds and yeasts were enumerated by surface plating 0.1 mL of the diluted sample in selective media. Baird-Parker Agar (BP; Oxoid) was used for coagulase-positive *Staphylococcus*, Mannitol Egg Yolk Polymyxin Agar (MYP, Oxoid) for presumptive *B. cereus* and Yeast Glucose Chloramphenicol Agar (YGC; Merck, Germany) for molds and yeasts. Plates were spread using a Drigalski spatula and incubated under aerobic conditions at 35 ± 1 °C for 48 ± 2 h (BP), 30 ± 1 °C for 18 to 24 h (MYP), and 25 ± 1 °C for 5 days (YGC).

*Escherichia coli* was enumerated using the pour plate method, where 1 mL of the diluted sample was transferred to a Petri dish, followed by the addition of Tryptone Bile X-glucuronide Agar (TBX; Neogen, USA). After homogenization, plates were incubated aerobically at 44 ± 1 °C for 18 to 24 h.

For the detection of *Salmonella* spp., BPW suspensions were pre-enriched at 37 ± 1 °C for 18 to 24 h. Subsequently, 0.1 mL and 1 mL aliquots were transferred to Rappaport-Vassiliadis Soy (RVS; Oxoid, Basingstoke, UK) and Muller-Kauffmann Tetrathionate Novobiocin (MKTTn; Oxoid) and incubated at 45 ± 1 °C and 37 ± 1 °C for 24 ± 3 h, respectively. For subsequent selective enrichment, 10 µL of the pre-enriched sample was streaked onto Xylose Lysine Deoxycholate Agar (XLD) and Brilliant Green Agar (BGA; Oxoid).

For mold and yeast enumeration, serial decimal dilutions were prepared in BPW and 0.1 mL of each dilution were surface plated onto YGC agar. All plates were analyzed for colonies displaying typical morphology of the target microorganism. Additionally, morphologically distinct colonies cultured on Nutrient Agar (NA; Neogen, USA) were selected and identified using Matrix-assisted Laser Desorption/ionization-Time of Flight mass spectrometry (MALDI-TOF MS).

### Isolation and identification of lactic acid bacteria (LAB)

LAB were isolated following a method adapted from ISO 15214:1998 [20]. Serial decimal dilutions of the samples were prepared in BPW and 0.1 mL were surface plated onto Man, Rogosa & Sharpe (MRS) agar using a Drigalski spatula. Plates were incubated anaerobically at 30 ± 2 °C for 48 ± 3 h.

Following incubation, colonies were selected based on morphological characteristics such as size, shape, color, texture, and margin, with up to 24 colonies recovered per sample. Isolates were purified in MRS broth and incubated under the same conditions prior to identification by MALDI-TOF MS.

### Antimicrobial susceptibility testing

Antimicrobial susceptibility test was performed using the disk diffusion method (Kirby-Bauer) and BrCAST reference. Prior to testing, LAB isolates and distinct colonies of *C. malonaticus* and *D. acidovorans* were cultured in MRS and BHI broths. The following antibiotics were tested: vancomycin (30 µg), tetracycline (30 µg), chloramphenicol (30 µg), amoxicillin (10 µg), gentamicin (120 µg), ampicillin (10 µg), streptomycin (300 µg), erythromycin (15 µg), ciprofloxacin (5 µg), and penicillin (10 µg).

Bacterial suspensions were adjusted to a 0.5 McFarland standard in 0.85% saline solution and spread onto the appropriate agar medium using a sterile swab. Antibiotic disks were placed to the agar surface, and plates were incubated either anaerobically at 30 ± 2 °C (MRS) or aerobically at 36 ± 2 °C for 24 h (BHI), according to the growth requirements of each isolate. Inhibition zone diameters were measured and interpreted as susceptible or resistant according to BrCAST criteria.

### Whole genome sequencing and genomic analysis

Five isolates were selected for whole-genome sequencing based on their antimicrobial susceptibility profiles: *Lactiplantibacilus plantarum*,* Levilactobacillus brevis*,* Leuconostoc mesenteroides*,* Enterococcus faecium* and *Cronobacter malonaticus*. Genomic DNA was extracted using the PureLink™ Genomic DNA Mini Kit and quantified using the Qubit™ dsDNA Quantification Assay Kit. Samples with DNA concentration ≥ 10 ng/µL were submitted to shotgun sequencing by IPEC Guarapuava (Brazil) using the Illumina NovaSeq 6000 platform with paired-end 150 bp reads.

Data analysis included an initial quality control step for the reads using the *fastp* (v0.23.2) [22], with the flags -q 30 -w 30 --cut_front --cut_tail --n_base_limit 0 --length_required 50. Genome assembly *de novo* was carried out using SPAdes software (v3.15.5) [23], applying the “--isolate” flag due to the high coverage obtained.

AMR genes were identified using the RGI (Resistance Gene Identifier, v6.0.0) tool against the CARD (Comprehensive Antibiotic Resistance Database, v3.2.6) [24], with main function and strict alignment thresholds and ≥ 85% identity of matching region. Mobile genetic elements and associated resistance genes were investigated using geNomad [25] with default parameters.

Sequence types (STs) were determined using the Multi-Locus Sequence Typing (MLST) database. The pathogenic potential of each genome was subsequently assessed using the PathogenFinder 1.1 web server, which predicts the probability of a bacterium being a human pathogen based on genomic features. Genomes with a predicted pathogenicity probability ≥ 0.5 were classified as potentially pathogenic.

## Results and discussion

### Identification of indicator and pathogenic microorganisms

None of the analyzed samples tested positive for *Salmonella* spp., *E. coli* or coagulase-positive *Staphylococcus* (Table 1). No presumptive *B. cereus* colonies were confirmed according to the ISO-based enumeration method. However, one isolate recovered during LAB isolation was classified as *B. cereus* by MALDI-TOF MS.

**Table 1:** Microbiological counts of artisanal cashew-based plant-based cheese analogues

Samples	Microorganisms				
	*Salmonella spp.*	*Escherichia coli*	coagulase positive* Staphylococcus*	*Bacillus cereus*	Molds and yeasts
	Detection in25 g		CFU/g		
S1	ND	< 10	< 100	< 100	> 300 × 10³
S2	ND	< 10	< 100	< 100	100
S3	ND	< 10	< 100	< 100	9.7 × 10⁴
S4	ND	< 10	< 100	< 100	4.6 × 10³
S5	ND	< 10	< 100	< 100	1.9 × 10⁴
S6	ND	< 10	< 100	< 100	< 100
S7	ND	< 10	< 100	< 100	< 100
S8	ND	< 10	< 100	< 100	< 100
S9	ND	< 10	< 100	< 100	> 300 × 10³
S10	ND	< 10	< 100	< 100	210 × 10³
S11	ND	< 10	< 100	< 100	8.4 × 10⁴
S12	ND	< 10	< 100	< 100	< 100
S13	ND	< 10	< 100	< 100	7.9 × 10³

*ND* Not detected

The absence of the main foodborne pathogens and hygiene indicators investigated suggests that the analyzed products were manufactured under satisfactory hygienic conditions. Similar findings were reported by Correia et al. (2026) [26], who found no evidence of *Salmonella* spp. or *Listeria monocytogenes* in commercial plant-based cheese analogues from Brazil, but detected *B. cereus* spores in 27% of the products evaluated. In the present study, although only one *B. cereus* isolate was detected and was not recovered through the selective enumeration procedure, its occurrence deserves attention because some strains can produce toxins associated with foodborne illness [27]. As this microorganism is commonly associated with plant-derived ingredients and environmental sources, its presence may reflect contamination originating from raw materials or processing environments [27], reinforcing the importance of monitoring spore-forming bacteria throughout the production chain of plant-based cheese analogues.

Most samples exhibited high mold and yeast counts, with predominance of yeasts (Table 1). According to Brazilian legislation (IN No. 313/2024), microbiological limits for molds and yeasts do not apply to foods intentionally produced with viable microorganisms. Therefore, this high counts in fermented plant-based cheese analogues should not necessarily be interpreted as indicators of poor microbiological quality. Several samples analyzed in this study declared the use of probiotic, mesophilic, or vegan cultures on their labels, which may partially explain the observed counts. In addition, undeclared microorganisms associated with spontaneous fermentation may also contribute to these microbial populations [5].

From a technological perspective, yeasts and molds may be desirable in fermented plant-based cheese analogues due to their contribution to flavour development during maturation. Species such as *Geotrichum candidum* are commonly used as ripening cultures because of their lipolytic and proteolytic activities, which can enhance sensory properties [10, 28]. However, in products not intended to fermentation or maturation processes, elevated yeast counts may indicate spoilage potential or insufficient process control.

### Isolation and identification of lactic acid bacteria (LAB)

**Fig. 1:**
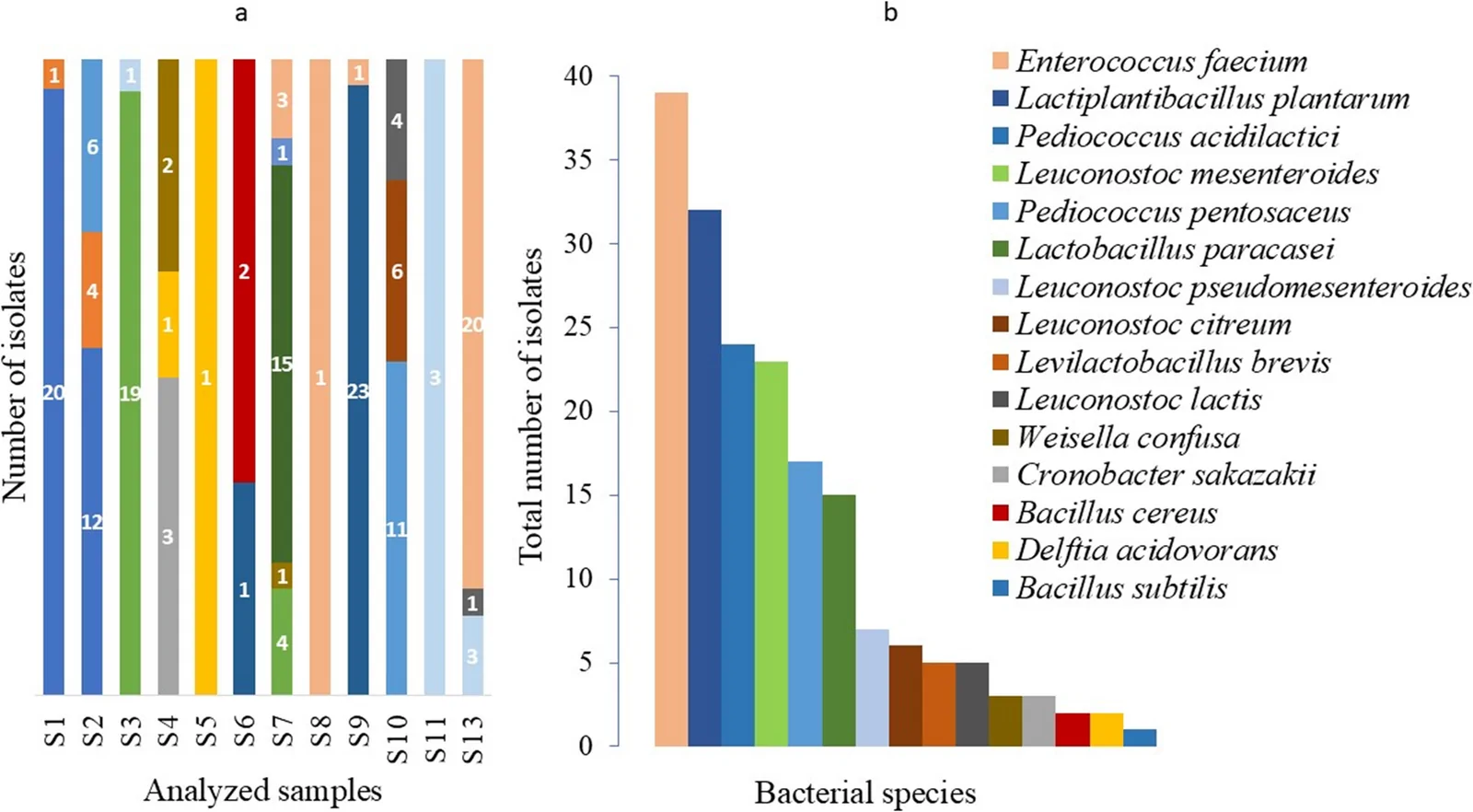
Distribution of bacterial isolates among the analyzed samples (**a**) and the total number of isolates identified for each species (**b**)

Presumptive LAB were detected in 10 of the 13 analyzed samples, yielding a total of 176 isolates, while no LAB were recovered from the other three samples. The distribution of bacterial species varied among products (Fig. 1A). Marked variation in bacterial community structure was observed across samples. Whereas some products were dominated by a single species, others contained a broader range of bacterial taxa. These patterns may reflect differences in raw materials, processing conditions, starter culture application, and the occurrence of spontaneous fermentation.

The LAB community was dominated by *E. faecium* (39 isolates), *L. plantarum* (32 isolates), *Pediococcus acidilactici* (24 isolates), *L. mesenteroides* (23 isolates), *Pediococcus pentosaceus* (17 isolates), and *Lacticaseibacillus paracasei* (15 isolates) (Fig. 1B). Additionally, a few non-LAB species were identified, namely *Bacillus subtilis*, *B. cereus*, *Cronobacter malonaticus*, and *Delftia acidovorans*.

Several of the predominant species identified in this study have previously been reported in fermented plant-based cheese analogues [16]. In particular, *L. plantarum*, *Leuconostoc* spp., and *Pediococcus* spp. are well adapted to plant-based substrates and play important roles in acidification, flavour development, and texture formation during fermentation [12, 29]. However, the predominance of *E. faecium* observed here differs from previous studies, in which *Lactococcus lactis* and *L. mesenteroides* were reported as the most abundant species [16]. Although *Enterococcus* spp. are commonly found in fermented foods and may contribute to fermentation processes [8], some strains have been associated with AMR and opportunistic infections [30]. Consequently, their high prevalence in the samples analyzed highlights the need for further strain-level characterization to assess their safety and technological relevance.

### Identification of other bacteria with potential pathogenicity

In addition to the target microorganisms investigated, atypical colonies recovered on BGA medium were identified as *C. malonaticus* and *Delftia acidovorans* (Fig. 1) in two samples (S4 and S5). Among these species, *C. malonaticus* warrants particular attention due to its opportunistic pathogenic potential and association with human infections. Members of the genus *Cronobacter* have been isolated from a wide variety of food products, including cereals, herbs, vegetables, dairy products, and powdered infant formula [31, 32]. Although the primary reservoirs of *Cronobacter* spp. remain poorly defined, plant-derived materials and food-processing environments are considered important sources of contamination. Furthermore, the capacity of these bacteria to form biofilms may enhance their persistence in processing facilities, thereby increasing the risk of contamination during food production [33, 34].

*Delftia acidovorans* is an environmental bacterium frequently detected in water systems and biofilms [35]. Although generally regarded as a low-virulence microorganism, it has occasionally been associated with opportunistic infections, particularly among immunocompromised individuals [36]. Its detection in the present study most likely reflects environmental contamination originating from raw materials or processing environments rather than a direct role in the fermentation process.

### Antimicrobial resistance

From 69 representative isolates obtained, 20 strains were selected for antimicrobial susceptibility testing. Resistance was detected in 11 isolates (55%), representing four bacterial species (Table 2). The resistance phenotypes were primarily associated with ciprofloxacin, tetracycline, gentamicin, and penicillin. Two *E. faecium* isolates exhibited resistance to gentamicin and ciprofloxacin, consistent with previous reports describing AMR among food-associated *Enterococcus* spp [30]. In contrast, *D. acidovorans* was susceptible to all antimicrobials tested, whereas *C. malonaticus* displayed resistance to penicillin.

**Table 2:** Antimicrobial susceptibility profiles of selected representative bacterial isolates

Isolate	Sample	Number of isolates	Number of isolates analyzed (%)	Antimicrobial resistance	Number of resistant isolates (%)
*Pediococcus pentosaceus*	S2	6	2 (33)	Ciprofloxacin	2 (100)
	S10	11	3 (27)	NI	0 (0)
*Pediococcus acidilactici*	S6	1	1 (100)	Tetracycline	1 (100)
				Ciprofloxacin	1 (100)
	S9	23	3 (13)	NI	0 (0)
*Enteroccocus faecium*	S7	3	2 (67)	Gentamicin	1 (50)
			2 (67)	Ciprofloxacin	1 (50)
	S8	1	1 (100)	NI	0 (0)
	S9	1	1 (100)	NI	0 (0)
	S13	20	3 (15)	Ciprofloxacin	3 (100)
*Cronobacter malonaticus*	S4	3	2 (67)	Penicillin	2 (100)
Total		69	20 (29)		11 (55)

*NI* Not identified

Previous studies have also documented resistance among LAB isolated from fermented foods, particularly to vancomycin, aminoglycosides, and quinolones [37]. These findings reinforce the importance of monitoring AMR in microorganisms associated with fermented food products.

### Genomic sequencing and resistome analysis

Whole-genome sequencing was performed on five representative isolates selected based on their antimicrobial susceptibility profiles. Among the sequenced strains, AMR genes were detected only in *C. malonaticus* genome. The identified resistance determinants, their associated antimicrobial classes, and the corresponding resistance mechanisms are presented in Table 3. *C. malonaticus* is a Gram-negative opportunistic pathogen known for its virulence potential and its ability to acquire and maintain AMR determinants [38, 39]. Most of the resistance genes detected in this species were associated with multidrug efflux systems and regulatory pathways, which may contribute to reduced susceptibility to multiple antimicrobial classes, including fluoroquinolones, cephalosporins, and tetracyclines, as well as disinfectants and antiseptic compounds.

**Table 3:** Antimicrobial resistance genes, antimicrobial classes, associated antibiotics, and resistance mechanisms detected in *Cronobacter malonaticus*

ARG	Antibiotic class	Antibiotic	AMR mechanisms
*CMA-2*	cephalosporin	benzalkonium chloride	antibiotic inactivation
*marA*	fluoroquinolone; monobactam; carbapenem; cephalosporin; glycylcycline; penicillin beta-lactam; tetracycline; rifamycin; phenicol; disinfecting agents and antiseptics	tigecycline; tetracycline; rifampin; chloramphenicol; ampicillin; cefalotin; triclosan	antibiotic efflux; reduced permeability to antibiotic
*emrR*	fluoroquinolone	nalidixic acid	antibiotic efflux
*emrB*	fluoroquinolone	nalidixic acid	antibiotic efflux
*rsmA*	fluoroquinolone; diaminopyrimidine; phenicol	trimethoprim; chloramphenicol	antibiotic efflux
*CRP*	macrolide antibiotic; fluoroquinolone; penicillin beta-lactam	erythromycin; cloxacillin; oxacillin; norfloxacin	antibiotic efflux
*Shigella flexneri acrA*	fluoroquinolone; cephalosporin; glycylcycline; penicillin beta-lactam; tetracycline; rifamycin; phenicol; disinfecting agents and antiseptics	tigecycline; ciprofloxacin; tetracycline; rifampin; chloramphenicol; ampicillin; cefalotin; triclosan	antibiotic efflux
*E. coli AcrAB-TolC* with *MarR* mutations conferring resistance to ciprofloxacin and tetracycline	fluoroquinolone; cephalosporin; glycylcycline; penicillin beta-lactam; tetracycline; rifamycin antibiotic; phenicol; disinfecting agents and antiseptics	tigecycline; ciprofloxacin; tetracycline; rifampin; chloramphenicol; ampicillin; cefalotin; triclosan	antibiotic target alteration; antibiotic efflux

Regarding resistance determinants located in plasmid-associated regions, genes related to vancomycin, chloramphenicol, fosfomycin, and tetracycline resistance were detected in *C. malonaticus* and *L. brevis* (Table 4). In *C. malonaticus*, most of the identified genes were associated with multidrug efflux systems and regulatory mechanisms commonly found in Gram-negative bacteria. In contrast, *L. brevis* harbored genes encoding tetracycline efflux transporters belonging to the Major Facilitator Superfamily (MFS).

**Table 4:** Plasmid-associated antimicrobial resistance genes identified in the sequenced isolates

	Bacteria			
	*Cronobacter malonaticus*			*Levilactobacillus brevis*
Contig	NODE_20	NODE_11		NODE_48
Length	55,793	131,011		11,767
Coverage	370.357707	381.537737		520.296920
Topology	DTR	DTR		No terminal repeats
No. genes	59	114		12
Genetic code	11	11		11
Plasmid score	1	1		9,999
FDR	NA	NA		NA
n hallmarks	3	4		0
Marker enrichment	262,956	378,914		20,608
Conjugation genes	NA	NA		NA
AMR genes	NF000402	NF033134	NF000496	NF012174
Annotation accessions	PF16968; K18941; TIGR02915; COG4567	K08162; COG2211	PF14506; COG3565; TIGR00068; K11210	PF00083; TIGR00903; K08221; COG2807
Annotation description	PEP-CTERM-box response regulator transcription factor	Na+/melibiose symporter or related transporter	*CppA* N-terminal	Major facilitator 4 family protein
NIH	Vancomycin resistance response regulator transcription factor *VanR-B*	*CmlA/FloR* family chloramphenicol efflux MFS transporter	Fosfomycin resistance glutathione transferase	*Tet(A)/Tet(B)/Tet(C)* family tetracycline efflux MFS transporter

*FDR* False discovery rate

Although intrinsic resistance is a recognized characteristic of several LAB species, the presence of AMR genes carried by mobile genetic elements in food-associated bacteria remains a concern due to their potential for horizontal dissemination [14, 17, 37, 40]. In this context, the detection of resistant microorganisms and resistance determinants in fermented plant-based foods reinforces the importance of integrating microbiological and genomic monitoring strategies, particularly for strains with potential technological or probiotic applications. Although the occurrence of resistance was limited among the isolates evaluated, the detection of these genes highlights the potential role of food-associated microorganisms as reservoirs of AMR determinants and underscores the value of combining phenotypic and genomic approaches for surveillance purposes.

The occurrence of resistance genes in plasmid-associated regions is particularly relevant because mobile genetic elements may facilitate the horizontal dissemination of AMR determinants within bacterial communities. Although transferability assays were not conducted in the present study, the genomic context of these genes highlights the need for further investigations into their mobility and dissemination potential in food-associated microbiota.

Pathogenicity prediction analyses revealed marked differences among the sequenced isolates. *C. malonaticus* exhibited a high predicted pathogenicity score (0.9559), whereas *L. brevis* showed a substantially lower probability (0.0374). These results are consistent with the recognized opportunistic pathogenic potential of *Cronobacter* species [31].

The detection of tetracycline resistance determinants, including *tet(A)*, *tet(B)*, and *tet(C)*, in *L. brevis* underscores the importance of evaluating AMR in microorganisms associated with fermented foods. Although this species is commonly found in fermented products and is generally regarded as safe [36], the presence of acquired resistance genes warrants attention when selecting strains for technological or probiotic applications. Collectively, these findings support previous reports indicating that food-associated bacteria, including LAB, may serve as reservoirs of AMR determinants and reinforce the importance of continued surveillance from a food safety perspective.

## Conclusions

This study is one of the firsts to investigate the microbiological safety and antimicrobial resistance of plant-based cheese analogues produced in Brazil, contributing to a better understanding of this emerging and relatively underexplored area. Overall, the analyzed products showed a low occurrence of classical foodborne pathogens and hygiene indicators, suggesting satisfactory microbiological quality. However, opportunistic bacteria of potential public health relevance, including *B. cereus* and *C. malonaticus*, were detected in a limited number of samples. AMR phenotypes were identified in a subset of isolates, and whole-genome sequencing revealed resistance-associated genes, including determinants located in plasmid-associated regions. Although the transferability of these genes was not evaluated, their occurrence highlights the importance of monitoring AMR in microorganisms associated with fermented plant-based foods. These findings reinforce the need for continued microbiological monitoring in fermented plant-based foods, particularly regarding AMR surveillance and the safety evaluation of microorganisms used for technological purposes. Further studies should investigate the transferability of resistance genes and the ecology of microbial communities associated with these emerging fermented foods.

## Supplementary Information

Below is the link to the electronic supplementary material.

Supplementary Material 1 (DOCX 464 KB)

## Data Availability

Sequence data that support the findings of this study have been deposited in the National Center for Biotechnology Information - NCBI GenBak under the BioProject ID PRJNA1406699.
