# Supplementary material for: Microbiological safety and antimicrobial resistance in cashew-based plant-based cheese analogues
Source: Braz J Microbiol. 2026 Sep 23;57(1):276. doi: 10.1007/s42770-026-02092-7 (PMC13601488; doi:10.1007/s42770-026-02092-7)
Supplement: Supplementary file 1 [file 42770_2026_2092_MOESM1_ESM.docx]

**Supplementary table**

**Supplementary Table 1.** Studied cashew-based plant-based cheese analogue samples.

| Origin | Producer | Sample | Identification | Ingredients |
| --- | --- | --- | --- | --- |
| Rio Grande do Sul State | A |  | S1 | NI |
|  |  |  | S2 | NI (smoked) |
|  | B |  | S3 | Coconut oil, cashew nut yogurt (cashew nuts, water, lactic ferment), sweet tapioca starch, potato flour, nutritional yeast, salt, vinegar, and sorbic acid preservative (smoked) |
| São Paulo State | C |  | S4 | Cashew nuts, water, turmeric, salt, coconut oil, natural smoke flavor, and yeast |
|  |  |  | S5 | Cashew nuts, coconut oil, yeast, salt, nutritional yeast, potato starch, seaweed extract, and sweet tapioca starch |
|  | D |  | S6 | Fresh cassava, cashew nuts, extra virgin olive oil, vegan culture, and salt (smoked) |
|  | E |  | S7 | Cashew nuts, water, Mossoró salt, and mesophilic culture |
|  |  |  | S8 | Cashew nuts, water, Mossoró salt, and mesophilic culture (smoked) |
|  | F |  | S9 | Cashew nuts, sesame seeds, water, probiotic culture, salt, and citric acid |
|  |  |  | S10 | Cashew nuts, water, probiotic culture, salt, citric acid, activated charcoal, and essential oil |
| Pernambuco State | G |  | S11 | Cashew nuts, water, salt, and probiotic |
|  |  |  | S12 | Cashew nuts, water, salt, and probiotic (smoked) |
| Distrito Federal, Federal Capital | H |  | S13 | Raw cashew nuts, water, coconut oil, salt, *Penicillium candidum*, and *Lactobacillus acidophilus* |

^Note: NI = not informed.^
